# Reduced axonal caliber and structural changes in a rat model of Fragile X syndrome with a deletion of a K-Homology domain of *Fmr1*

**DOI:** 10.1038/s41398-020-00943-x

**Published:** 2020-08-12

**Authors:** Carla E. M. Golden, Yohan Yee, Victoria X. Wang, Hala Harony-Nicolas, Patrick R. Hof, Jason P. Lerch, Joseph D. Buxbaum

**Affiliations:** 1 Department of Psychiatry, Icahn School of Medicine at Mount Sinai, New York, NY USA; 2 Seaver Autism Center for Research and Treatment, Icahn School of Medicine at Mount Sinai, New York, NY USA; 3 Department of Medical Biophysics, University of Toronto, Toronto, ON Canada; 4 Mouse Imaging Centre, The Hospital for Sick Children, Toronto, ON Canada; 5 BioMedical Engineering and Imaging Institute, Icahn School of Medicine at Mount Sinai, New York, NY USA; 6 Nash Family Department of Neuroscience, Icahn School of Medicine at Mount Sinai, New York, NY USA; 7 Friedman Brain Institute, Icahn School of Medicine at Mount Sinai, New York, NY USA; 8 Mindich Child Health and Development Institute, Icahn School of Medicine at Mount Sinai, New York, NY USA; 9 Wellcome Centre for Integrative Neuroimaging, FMRIB, Nuffield Department of Clinical Neuroscience, University of Oxford, Oxford, UK; 10 Department of Genetics and Genomic Sciences, Icahn School of Medicine at Mount Sinai, New York, NY USA

**Keywords:** Neuroscience, Autism spectrum disorders

## Abstract

Fragile X syndrome (FXS) is a neurodevelopmental disorder that is caused by mutations in the *FMR1* gene. Neuroanatomical alterations have been reported in both male and female individuals with FXS, yet the morphological underpinnings of these alterations have not been elucidated. In the current study, we found structural changes in both male and female rats that model FXS, some of which are similarly impaired in both sexes, including the superior colliculus and periaqueductal gray, and others that show sex-specific changes. The splenium of the corpus callosum, for example, was only impaired in males. We also found reduced axonal caliber in the splenium, offering a mechanism for its structural changes. Furthermore, we found that overall, male rats have higher brain-wide diffusion than female rats. Our results provide insight into which brain regions are vulnerable to a loss of *Fmr1* expression and reveal an impairment at the level of the axon that could cause structural changes in white matter regions.

## Introduction

Fragile X syndrome (FXS) is a monogenic disorder caused by mutations in the *FMR1* gene, which encodes fragile X mental retardation protein (FMRP). It is the leading monogenic cause of autism spectrum disorder (ASD), the most frequent known form of inherited intellectual disability (ID), is often comorbid with attention-deficit/hyperactivity disorder (ADHD), and can cause sensory hyperarousal^1,2^. With the use of magnetic resonance imaging (MRI), alterations in brain structure have been identified in both gray and white matter regions in individuals with FXS and have been associated with aberrant cognitive phenotypes. To understand how a lack of functional Fmrp leads to these deficits, animal models of FXS have been generated. Compared to other classes of animals and in vitro models, rodents are superb models for studying the specific biological processes that are perturbed following a loss of Fmrp. Unlike nonhuman primates which are evolutionarily closer to humans, rodent models are bred on isogenic background lines and produce larger litters, allowing for the manipulation of one gene at a time and larger sample sizes. Unlike cell lines and invertebrates, rodent behaviors can be mapped to human analogs that are found to be impaired in FXS. In the FXS population, the skewed sex ratio (4 males to 1 female) is attributed to the sex difference in the relative amount of Fmrp expressed. An added benefit of using rodent models is that homozygous females can be produced and compared to transgenic males to see if there are sex differences that follow from a loss of Fmrp expression when both sexes do not express Fmrp. Critically, the integrity of brain regions and the white matter that connects them has not been directly assessed in a rat model of FXS. The regions that should be given priority in studies of FXS rat models have therefore not been elucidated. As a result, the mechanism by which a lack of Fmrp causes structural alterations in the rat brain is poorly understood.

In FXS, the white matter pathways that connect frontal and striatal regions are impaired in development^3^. It is hypothesized that these alterations underlie the reported deficits in attention in FXS. However, other regions and white matter tracts that are also implicated in attention show anatomical alterations in FXS, such as the thalamus, internal capsule, and the splenium of the corpus callosum^4^. Structural deficits in white matter have also been identified in *Fmr1* knockout (KO) mice and resemble those seen in individuals with FXS^5–7^. Importantly, this includes regions known to be recruited during visuospatial attention, namely, the superior colliculus, the splenium of the corpus callosum, and the white matter of the medial prefrontal cortex. There are also deficits in resting state functional connectivity in individuals with FXS and *Fmr1* KO mice^8,9^. One example of this is hypoconnectivity in the somatosensory network of *Fmr1* KO mice, again suggesting an anatomical link between decreased Fmrp expression and deficits in sensory perception^9^. We predict that similar regions will be impaired in a rat model of FXS.

FMRP could play a role in structural integrity through its repression of protein translation of mRNA targets^10^. In FXS, without FMRP, the protein expression of these mRNA targets is elevated because their translation is no longer repressed. Many of the mRNA targets of Fmrp encode key presynaptic and postsynaptic proteins that affect synapse development, including cytoskeleton scaffolding and remodeling, as well as transcripts involved in the development of myelin^1^. Dysregulation of their expression in the absence of FMRP is thought to contribute to the heightened levels of long, immature dendritic spines, and increased spine density that is commonly seen in the pyramidal neurons of individuals with FXS and animal models^1^. Therefore, these alterations in neurons and oligodendrocytes could influence the brain’s functional connectivity; however, a mechanism by which this might occur has not yet been elucidated. Thus, it is pertinent to perform a brain-wide, unbiased study of structural integrity in rats.

We recently identified a sustained attention deficit that presented in both sexes of a rat model of FXS that has a deletion of an mRNA-binding domain called the K-homology 1 (KH1) domain^11^. We refer to this rat as the *Fmr1-*^*Δ*^*exon 8* rat. Instead of Fmrp, the *Fmr1-*^*Δ*^*exon 8* rat expresses Fmrp-^Δ^KH1 at low levels. This rat model also has been shown to have impaired cortical representation of speech sounds^12^. Here, we assess neuroanatomical integrity in this *Fmr1-*^*Δ*^*exon 8* rat model of FXS. We used structural MRI to measure regional volumes and tissue densities, diffusion tensor imaging (DTI) to assess white matter integrity, and electron microscopy to uncover deficits in ultrastructure that could reflect functional perturbations. Both male and female *Fmr1-*^*Δ*^*exon 8* rats were used in these studies to be able to test for an effect of sex. Heterozygous rats, a phenomenon only possible in females, were included to enable the assessment of a dose-dependent effect of Fmrp-^Δ^KH1.

## Materials and methods

### Experimental design

The objective of this controlled laboratory experiment was to use an unbiased approach to identify brain regions that are affected by a loss of *Fmr1* expression and then determine the mechanism that could underlie this change. We hypothesized that brain regions that are affected in individuals with FXS would also be altered in the *Fmr1-*^*Δ*^*exon 8* rat model of FXS. Sample sizes used in the MRI experiments were determined based on a previous study of a rodent model of autism^13^ and sample sizes used for the electron microscopy experiments matched similar studies. Sex was considered as a variable of interest and its effect was reported where meaningful. Analysis of the MRI data was replicated with two different analytical pipelines and electron microscopy was replicated in two separate cohorts, each constituting a sample size of three. The degree to which the MRI results were validated in the second analysis are detailed in the “Results” section and the findings from the electron microscopy experiments were substantiated in the second cohort. During image acquisition, manual quality control of the MRI segmentations, and tracing of the electron microscopy images, the experimenters were blind to genotype. Each manually performed operation was validated across two experimenters to limit subjectivity. Each experimental animal was scanned in the MRI on the same day as a same-sex control animal. Images with obvious artifacts or masks that did not align to the image were excluded before the statistical analyses commenced. Individual data points that were outside 1.5 times the interquartile range were omitted from the analysis. No outliers were included in the results reported.

### Generation of the *Fmr1-*^*Δ*^*exon 8* rat model

The *Fmr1-*^*Δ*^*exon 8* rat model was generated using zinc finger nucleases (ZFNs) in the outbred Sprague-Dawley background by Horizon Labs (Boyertown, PA USA) as previously described^11^.

### Animal breeding, care, and husbandry

This study used age-matched male and female littermate rats. To produce both male genotypes (*Fmr1-*^*Δ*^*exon 8*^+/y^ (WT) and *Fmr1-*^*Δ*^*exon 8*^−*/*y^) and all three female genotypes (*Fmr1-*^*Δ*^*exon 8*^+/+^ (WT)*, Fmr1-*^*Δ*^*exon 8*^+/−^, and *Fmr1-*^*Δ*^*exon 8*^−/−^), we set up two pairs of breeders: WT male × *Fmr1-*^*Δ*^*exon 8*^+/−^ and *Fmr1-*^*Δ*^*exon 8*^−/y^ × *Fmr1-*^*Δ*^*exon 8*^+/−^. All five groups were used for the MRI experiments and WT and *Fmr1-*^*Δ*^*exon 8*^−/y^ male rats were used for the electron microscopy experiments at 8–10 weeks old (see Supplementary Table 1 for groups and sample sizes used in each analysis). All rats were kept under veterinary supervision in a 12 h reverse light/dark cycle at 22 ± 2 °C. Animals were pair-caged with food and water available *ad libitum*. All animal procedures were approved by the Institutional Animal Care and Use Committee at the Icahn School of Medicine at Mount Sinai.

### MRI

All imaging was performed by the BioMedical Molecular Imaging Institute using a Bruker Biospec 70/30 7 T scanner with a B-GA12S gradient insert (gradient strength 440 mT/m and slew rate 3444 T/m/s). A Bruker 4-channel rat brain phased array was used for all data acquisition in conjunction with a Bruker volume transmit 86-cm coil. All rats (*N* = 15/group) were imaged on a heated bed and respiration was monitored continuously until the end of the scan. The animal was anesthetized using isoflurane anesthesia (3% induction and 1.5% maintenance). After a three-plane localizer, a field map was acquired and the rat brain was shimmed using Mapshim software. A DTI protocol was acquired with a Pulsed Gradient Spin Echo—Echo-planar imaging (EPI) sequence with the following parameters: repetition time (TR) = 5000 ms, echo time (TE) = 22.6 ms, 4 segments, 30 gradient directions with *b*-value = 1000 s/mm^2^ and 5 B0’s, field of view (FOV) = 25 mm, matrix = 128 × 128, slice thickness = 1 mm, skip = 0, 6 averages, total acquisition time = 1 hr. The voxel size was 0.195 × 0.195 × 1 mm^3^ (1000 µm-thick). A high resolution T2-weighted anatomical scan was obtained for 53 min with a 3D Rapid Acquisition with Relaxation Enhancement (RARE) sequence with a RARE factor of 8, TR = 777 ms, effective TE = 52 ms, FOV = 30 mm × 27.25 mm × 30 mm, matrix size 256 × 256 × 128. The voxel size was 0.117 × 0.117 × 0.234 mm^3^ (234 µm-thick).

### Semi-automated MRI region-based analytical pipeline

An MRI processing pipeline was used to perform semi-automated nonbiased brain segmentation, while blinded to genotype (*N* = 15/group)^14^. The pipeline is composed of six major steps: rigid registration of images to each other, generation of a whole-brain mask for each image, averaging of all images, creation of a whole-brain mask for this averaged image, segmentation of the average mask into regions of interest (ROIs), parcellation propagation of the segmented mask to individual subjects, and ROI-based statistics for the individual images. The deformation necessary to warp each subject’s image to the average was used to calculate the volume of the ROIs. After each mask was generated, it was improved manually in ITK-SNAP (www.itksnap.org)^15^. Segmentation into ROIs was performed using a template that was previously hand-segmented into 32 brain regions, listed in Supplementary Table 2.

Segmented masks for the individual images that did not closely match the segmented average mask (one *Fmr1-*^*Δ*^*exon 8*^−/−^ T2 mask, two female WT T2 masks, and one male WT DTI mask) and individual data point outliers, defined as being outside 1.5 times the interquartile range, were excluded from the analysis. Males and females were analyzed in two separate pipelines because the difference in their brain size would skew the averaging step such that it would be misaligned. The unsegmented brain masks were used to determine whole-brain measures. The volumes of each ROI and the whole brain were measured from the T2 images and mean voxel intensities of each ROI and the whole brain were measured from both the T2 and DTI images. Mean voxel intensities in the T2 images were used to determine tissue density where a lower mean intensity suggests denser tissue. The olfactory bulb, cerebellum, and brainstem were not included in the DTI analysis because the DTI images did not capture the entirety of these regions. Furthermore, regions without any known white matter were excluded from the DTI analysis, including the aqueduct, periaqueductal gray (PAG), and third, fourth, and lateral ventricles. In the analysis of the male-only data, for each independent variable, if the distribution was nonparametric according to the Shapiro–Wilk’s test, a Mann–Whitney *U* test was administered and if the distribution was normally distributed, a two-way ANOVA was applied. In the analysis of the female-only data, if the distribution was nonparametric, a Kruskal–Wallis test was administered, which was followed by a Dunn’s test to compare the individual means and an adjustment of the *p* values with the False Discovery Rate method, and if the data was parametric, an ANOVA was applied and followed by a post-hoc Tukey HSD test that compared the pairs of means and adjusted the *p* values to account for the additional comparisons. Due to the fact that many comparisons were made across ROIs, we applied a Bonferroni correction for multiple comparisons, the most conservative method of this nature. Genotype was the only between-groups factor. Custom scripts written in the R statistical programming environment were used for the statistical analysis (R Development Core Team, 2006).

### Automated MRI region-based and voxel-based analytical pipeline

T2 images were aligned together in an iterative registration procedure using the PydPiper framework that included a series of linear and nonlinear alignment steps, as previously described^16^. Briefly, images were first linearly aligned (6 degrees of freedom: rotations and translations) to a model template, so that all images were roughly in the same space and roughly in the same orientation. These images were then linearly aligned (12 degrees of freedom: rotations, translations, scaling, and shearing) to each other in a pairwise manner; each image was resampled with its average transformation to the other images. The linearly transformed resampled images were then averaged to create a linear (12-parameter) template. After creation of a linear template, images were then nonlinearly aligned with Advanced Normalization Tools (ANTs)^17^ (www.picsl.upenn.edu/software/ants) to this template and averaged to create a new updated template. Nonlinear alignment and averaging were repeated for a total of three iterations. The output of this pipeline included a final study-specific nonlinear average template, transformations mapping each image to the template, and voxel-wise Jacobian determinants corresponding to each transformation that represented the extent to which each image locally deformed to match the template. To identify voxels that were altered in volume, a *p* value significance was set to a voxel-wise false discovery rate (FDR)-corrected *p* value < 0.1. We controlled for the FDR instead of applying the Bonferroni method in the analysis of the voxel-wise data because the dataset was much larger and the Bonferroni method could therefore yield false-negatives.

DTI metrics were extracted for each subject from the raw imaging data with DTIFit (Functional Magnetic Resonance Imaging of the Brain Software Library). The raw T2 (no diffusion weighting, “S0”) images were registered together using the same pipeline described above. This DTI S0 template was further aligned to the T2 template generated in the steps above using ANTs, and concatenated transformations mapping each raw image to the T2 template were computed. The remaining images (fractional anisotropy (FA), mean diffusion (MD), axial diffusion (AD), radial diffusion (RD)) were resampled to the T2 template with their respective concatenated transforms to allow both voxel-wise and ROI analyses.

This T2 template was aligned to the T2 template that was used for the semi-automated analysis using ANTs in order to segment it into ROIs (see Supplementary Table 2) and resampled to the DTI templates generated in the automated procedure. For T2, volumes for each subject were computed by summing the voxel volumes under each ROI, weighted by the Jacobian determinants. This was done using the RMINC software library in R (https://github.com/Mouse-Imaging-Centre/RMINC). Mean volume for each ROI and each DTI metric were computed in T2 space using RMINC. Whole-brain values for the DTI metrics were calculated by summing the average metrics per ROI, weighted by volume of each ROI. The regional volume of the PAG was computed using the Waxholm Space Atlas (NITRC^18^, version two^19^), a better estimate of this region, once volumetric changes in voxels within it were identified.

For this analysis, *Fmr1-*^*Δ*^*exon 8*^−/−^ and *Fmr1-*^*Δ*^*exon 8*^*−/y*^ rats were compared to their WT male and female littermates (*N* = 15/group) so that the effect of sex could be examined. Data points that were outside 1.5 times the interquartile range were determined to be outliers and removed from the analysis. Statistical analysis began with the assessment of normality. Non-parametric data were evaluated with a linear model (LM) and parametric data were assessed with a two-way ANOVA, both with genotype and sex as factors. A Tukey HSD post hoc test was administered if there was a main effect of genotype or sex to correct for post hoc multiple comparisons. The output was corrected for multiple comparisons with a Bonferroni correction, just as in the semi-automated ROI-based analysis.

### Electron microscopy

Preparation for electron microscopy was performed by the Icahn School of Medicine’s Microscopy CoRE using protocols optimized to study the ultrastructure of nervous tissue. Male rats (WT: *N* = 7, *Fmr1-*^*Δ*^*exon 8*^*−/y*^: *N* = 6) were anesthetized and perfused using a peristaltic pump at a flow rate of 35 ml/min with 1% paraformaldehyde/phosphate buffered saline (PBS), pH 7.2, and immediately followed with 2% paraformaldehyde and 2% glutaraldehyde/PBS, pH 7.2, at the same flow rate for an additional 10–12 min. The brain was removed and placed in immersion fixation (same as above) to be postfixed for a minimum of one week at 4 °C. Fixed brains were sectioned using a Leica VT1000S vibratome (Leica Biosystems, Buffalo Grove, IL) and coronal slices (325 µm) containing the splenium of the corpus callosum were removed and embedded in EPON resin (Electron microscopy Sciences (EMS), Hatfield, PA). Briefly, sections were rinsed in 0.1 M sodium cacodylate buffer (EMS), fixed with 1% osmium tetroxide followed with 2% uranyl acetate, dehydrated through ascending ethanol series (beginning with 25% up to 100%), and infiltrated with propylene oxide (EMS) and then EPON resin (EMS). Sections were transferred to beem capsules and heat-polymerized at 60 °C for 72 h. Semithin sections (0.5 and 1 µm) were obtained using a Leica UC7 ultramicrotome, counterstained with 1% Toluidine Blue, coverslipped and viewed under a light microscope to identify and secure the region of interest. Ultrathin sections (80 nm) were collected on copper 300 mesh grids (EMS) using a Coat-Quick adhesive pen (EMS), and serial sections were collected on carbon-coated slot grids (EMS). Sections were counterstained with 1% uranyl acetate followed with lead citrate.

Sections were then imaged on a Hitachi 7000 electron microscope (Hitachi High-Technologies, Tokyo, Japan) using an advantage CCD camera (Advanced Microscopy Techniques, Danvers, MA). Ten areas per section from within the region of the splenium that contained cross sections of axons were chosen randomly to be imaged at 15,000× magnification. Images were adjusted for brightness and contrast using Adobe Photoshop CS4 11.0.1. Using ImageJ (www.imagej.nih.gov), 200 myelinated, and 200 unmyelinated axons were traced from each sample. The caliber of the myelinated and unmyelinated axons was defined as the area within the trace of the cell walls. The thickness of the myelin was calculated by subtracting the traces of the myelinated axons from traces that included the myelin (excluding perfusion artifacts). The number of myelinated axons per image was determined by counting by hand. The g-ratio was calculated as the caliber of each myelinated axon divided by its caliber plus the thickness of its myelin. The interaction between genotype and caliber was assessed. The amount of the total area in the image that was covered by the myelinated axons was calculated per image by multiplying the number of myelinated axons by the average caliber of the myelinated axons in the image and then comparing the result as a percentage of the total area of the image (58 253 186 nm^2^). Two cohorts of littermates were assessed where the first cohort had three WT and three *Fmr1-*^*Δ*^*exon 8*^*−/y*^ rats and the second cohort had four WT and three *Fmr1-*^*Δ*^*exon 8*^*−/y*^ rats. A Kolmogorov–Smirnov test was used to test for the distance between the empirical distribution functions of axon caliber. Data points that were outside 1.5 times the interquartile range for each rat were removed. A linear mixed model where the image file was nested in the rat ID, which was then nested in the cohort number was used to compute the effect of genotype with custom scripts written in the R statistical programming environment (R Development Core Team, 2006).

## Results

### Brain region volumes are altered in *Fmr1-*^*Δ*^*exon 8* rats

**Fig. 1:**
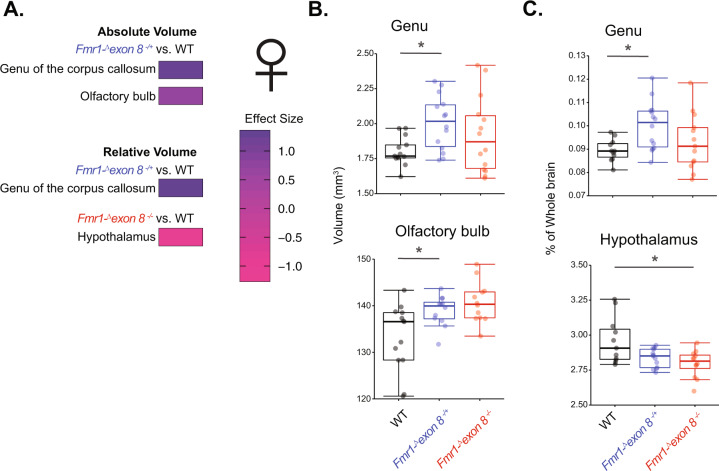
Absolute and relative volume of ROIs in female *Fmr1-*^*Δ*^*exon 8*^−/−^, *Fmr1-*^*Δ*^*exon 8*^*−*^^*/*^^*+*^, and WT littermates. **a** Heatmap of the effect sizes of absolute and relative volumes significantly affected by genotype in T2 images where an increase in dark purple denotes an increase in effect size compared to WT and pink denotes a decrease. **b** Boxplots of the regions that significantly changed in absolute volume, the genu, and olfactory bulb. **c** Boxplots of the regions that significantly changed in relative volume compared to the whole brain, the genu, and hypothalamus. Significance bars represent pair-wise comparisons from either a Tukey HSD or Dunn’s test, (WT: *N* = 13; *Fmr1-*^*Δ*^*exon 8*^*−*^^*/*^^*+*^: *N* = 15; *Fmr1-*^*Δ*^*exon 8*^−/−^: *N* = 14), *p < 0.05.

Structural T2-weighted MRI was used to assess both volume and white versus gray matter density in male and female *Fmr1-*^*Δ*^*exon 8* rats and their WT littermates. An effect of genotype on these measures across the whole brain and in 32 ROIs (see Supplementary Tables 2 and 3) was quantified. In a semi-automated analysis, ROIs were first determined based on a template (see “Methods”) aligned to an averaged image that was calculated from all subjects. This method of segmentation was then applied to each individual image. Given that male rodents have larger brains than female rodents^20^ because of their larger body size, the averaging step in this pipeline would create an average image that would be unrepresentative of both sexes. We therefore analyzed the males and females separately. In *Fmr1-*^*Δ*^*exon 8*^*−*^^*/*^^*y*^ (male) rats, the absolute volume of the superior colliculus was increased compared to their controls (Supplementary Fig. 1; ANOVA, *p* = 0.011). In female rats, there was a significant effect of genotype on the absolute volume of the genu of the corpus callosum and the olfactory bulb, a region that has relatively high Fmrp expression^21^ (Fig. 1a, b; Kruskal–Wallis, *p* = 0.047 and *p* = 0.01, respectively). Both regions were significantly larger in the *Fmr1-*^*Δ*^*exon 8*^*−*^^*/*^^*+*^ rats compared to their female WT littermates (Dunn’s test, *p adj*. = 0.02 and *p adj*.= 0.023, respectively). The increase in volume of the genu was not due to a change in whole-brain volume because it also increased in relative volume (i.e., percentage of total brain volume) (Fig. 1a, c; ANOVA followed by Tukey HSD, *p adj*. = 0.03). The hypothalamus was also reduced in volume relative to the whole brain in the *Fmr1-*^*Δ*^*exon 8*^−/−^ rats compared to female WT littermates (Fig. 1a, c; Kruskal–Wallis followed by Dunn’s test, *p adj*. = 0.02). This suggests that Fmrp can play a role in the development of both gray and white matter structures in male and female rats.

**Fig. 2:**
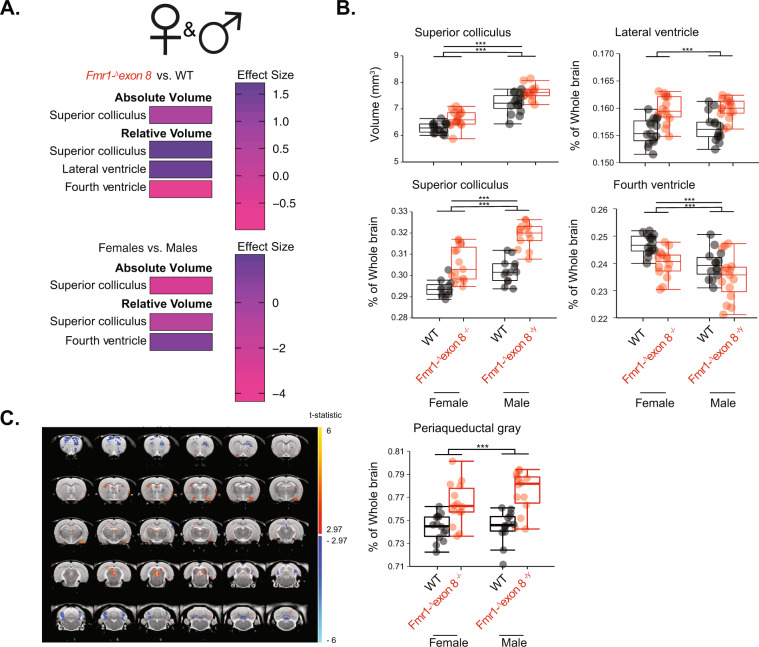
Absolute and relative brain region volumes of male and female WT and *Fmr1-*^*Δ*^*exon 8* littermates. **a** Heatmap of the effect sizes of absolute and relative volumes significantly affected by genotype after being Bonferroni-corrected in T2 images where an increase in dark purple denotes an increase in effect size in *Fmr1-*^*Δ*^*exon 8* compared to WT and females compared to males and pink denotes a decrease. **b** Boxplots of the regions that significantly changed in absolute and relative volumes, the superior colliculus and lateral and fourth ventricles. **c** Map of the t-statistic across the brain showing the voxels that are increased in volume in the *Fmr1-*^*Δ*^*exon* rats compared to WT rats and boxplots of mean relative volume of the PAG. Significance bars represent pair-wise comparisons from a Tukey HSD test, (*N* = 15/group), ***p < 0.001 and **p < 0.01.

In order to determine whether sex influences the development of these regions, we next validated our findings with a second, fully automated pipeline with the same division of ROIs (see Supplementary Table 2 for ROIs and Supplementary Table 3 for all data). This pipeline combined males and females in a single registration pipeline that was followed by the determination of ROIs for each subject individually. *Fmr1-*^*Δ*^*exon 8*^*−*^^*/*^^*+*^ rats were excluded so that there was an equal number of genotypes represented per sex (WT and *Fmr1-*^*Δ*^*exon 8* rats). This analysis validated that the absolute volume of the superior colliculus was increased in *Fmr1-*^*Δ*^*exon 8* rats, this time in male and female *Fmr1-*^*Δ*^*exon 8* rats pooled together (Fig. 2a, b; LM followed by Tukey HSD, *p adj*. = 4.67 × 10^−5^). It also identified an increase in the relative volume of the superior colliculus in male and female *Fmr1-*^*Δ*^*exon 8* rats (Fig. 2a, b; LM followed by Tukey HSD, *p adj*. = 1.59 × 10^−10^), suggesting that this difference was not due to changes in overall brain volume. In addition, likely due to subtle volumetric changes in the regions that surround them, the relative volume of the lateral ventricle increased and the relative volume of the fourth ventricle decreased in *Fmr1-*^*Δ*^*exon 8* rats (Fig. 2a, b; two-way ANOVA followed by Tukey HSD, *p adj*. = 2.01 × 10^−7^ and *p adj*. = 2.11 × 10^−4^, respectively). There was also a significant effect of sex on absolute and relative volume of the superior colliculus and relative volume of the fourth ventricle (Fig. 2a, b; LM followed by Tukey HSD, *p adj*. = 4.4 × 10^−13^ and *p adj*. = 5.08 × 10^−8^ and two-way ANOVA followed by Tukey HSD, *p adj*. = 1.43 × 10^−4^ respectively). However, this is because male brains were significantly larger than female brains (Supplementary Fig. 2A and B; LM followed by Tukey HSD, *p adj*. < 1.0 × 10^−20^) with a nominal *p* value that survives a Bonferroni correction. Therefore, these findings suggest that a reduction in *Fmr1* expression affects the volume of the superior colliculus in both male and female rats.

### Voxelwise volume changes in the superior colliculus and PAG in *Fmr1-*^*Δ*^*exon 8* rats

This pipeline transforms each image to a study-based average and thereby puts voxels across subjects in correspondence. Consequently, we were also able to test for local effects at the voxel level that would not otherwise be captured at the structural level. By applying independent linear models at each voxel that modeled volume change (i.e. the Jacobian determinant) as a function of sex, genotype, and their interaction, we found genotype-related effects in the periaqueductal gray and reproduced the ROI-based genotype effect in the superior colliculus. Specifically, many voxels within the superior colliculus and the PAG showed local volume increases in *Fmr1-*^*Δ*^*exon 8* rats relative to the control group (Fig. 2c; *p*FDR < 0.1). In the case of the superior colliculus, the volume increases seemed to be localized to the superficial layers, suggesting that the volume increase seen at the ROI level results from microscopic changes in this subregion. For the PAG, since the manual segmentation did not capture these volumetric changes, we used the Waxholm Space Atlas definitions of regions to confirm that the local volume changes proximal to the PAG are reflected at the level of the entire PAG region (the difference between the two segmentations and their template images are captured in Supplementary Fig. 3). At the ROI level, we found that there was a main effect of genotype (after a Bonferroni correction) on the relative volume of the PAG where the *Fmr1-*^*Δ*^*exon 8* rats had an increased volume (Fig. 2c; two-way ANOVA followed by Tukey HSD, *p adj*. = 2 × 10^−7^). The increase in size of the PAG could be the reason the fourth ventricle decreased in regional volume. There was no effect of sex on the relative volume of the PAG (*p* = 0.267). This voxelwise analysis provides specificity in identifying the local areas of the brain that change in structure due to a loss of intact Fmrp.

### Diffusion is altered in male and female *Fmr1-*^*Δ*^*exon 8* rats compared to sex-matched controls

We used DTI to evaluate white matter integrity, as white matter structure is affected in individuals with FXS. Specifically, we examined axial, radial, and mean diffusion (AD, RD, and MD, respectively) and total FA as measures of possible changes. FA is enhanced when parallel diffusivity is facilitated and/or perpendicular diffusivity is restricted, RD is increased following axonal and myelin damage, and AD is reduced after axonal damage^22,23^. Furthermore, MD may reflect pathology if it is increased in white matter.

**Fig. 3:**
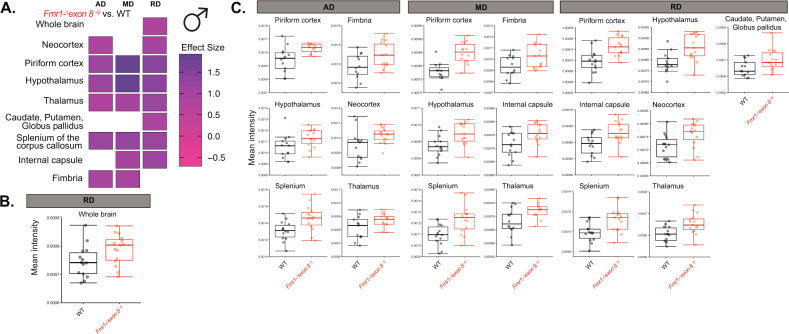
Diffusion indices in male *Fmr1-*^*Δ*^*exon 8*^*−*^^*/*^^*y*^ and WT littermates. **a** Heatmap of effect sizes of genotype on AD, MD, and RD where an increase in purple denotes an increase in *Fmr1-*^*Δ*^*exon 8*^*−*^^*/*^^*y*^ compared to WT rats and pink denotes a decrease. **b** Boxplot of mean intensity of RD being significantly increased across the whole brain in *Fmr1-*^*Δ*^*exon 8*^*−*^^*/*^^*y*^ rats. **c** Boxplots of the brain regions with significantly increased mean intensity of AD, MD, and RD in *Fmr1-*^*Δ*^*exon 8*^*−*^^*/*^^*y*^ rats, (WT: *N* = 14, *Fmr1-*^*Δ*^*exon 8*^*−*^^*/*^^*y*^: *N* = 15).

Similar to the T2 region-based analysis, we first analyzed the effect of genotype on these measures across the whole brain and 32 ROIs (see Supplementary Tables 2–4) in male and female rats separately in a semi-automated analysis. Amongst the male rats, RD was increased across the whole brain of *Fmr1-*^*Δ*^*exon 8*^*−*^^*/*^^*y*^ rats compared to WT littermates, suggesting brain-wide deficits in myelin and axon density (Fig. 3a, b; ANOVA, *p* = 0.043). This could be due to RD being increased in many forebrain regions, including the largest region of the brain in our analysis, the neocortex (Fig. 3a, c; ANOVA, *p* = 0.015). RD also increased in the piriform cortex, hypothalamus, and thalamus (Fig. 3a, c; ANOVA, *p* = 0.0009, *p* = 0.0017, and *p* = 0.0047, respectively), as well as the caudate, putamen, and globus pallidus (Fig. 3a, c; ANOVA, *p* = 0.032), the three of which were grouped together as one region in our analysis. Complementary to this increase in RD, there were also increases in MD in the forebrain in the piriform cortex, hypothalamus, and thalamus (Fig. 3a, c; ANOVA, *p* = 0.00012, *p* = 0.0056, and *p* = 0.02, respectively). Interestingly, AD, although often inversely proportional to RD and MD, also increased in many forebrain regions. AD was higher in the neocortex, piriform cortex, hypothalamus, and thalamus (Fig. 3a, c; ANOVA, *p* = 0.044, *p* = 0.0067, *p* = 0.039, and *p* = 0.047, respectively). While there were no alterations in these indices in the brainstem regions, there were changes in white matter pathways, as we predicted. All three diffusion indices, AD, MD, and RD, increased in the splenium of the corpus callosum (Fig. 3a, c; ANOVA, *p* = 0.0052, *p* = 0.003, and *p* = 0.0035). MD and RD were also enhanced in the internal capsule (Fig. 3a, c; ANOVA, *p* = 0.019 and *p* = 0.0065) and AD and MD were greater in the fimbria (Fig. 3a, c; ANOVA, *p* = 0.029 and *p* = 0.028). The whole-brain increase in RD and the further deficits in white matter regions suggests a brain-wide deficit in myelin and axonal projections following a loss of *Fmr1* expression in male rats.

**Fig. 4:**
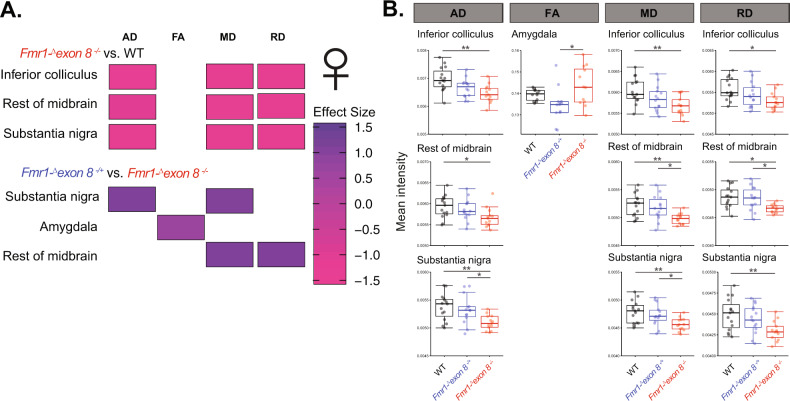
Diffusion indices in female *Fmr1-*^*Δ*^*exon 8*^−/−^, *Fmr1-*^*Δ*^*exon 8*^*−*^^*/*^^*+*^, and WT littermates. **a** Heatmap of effect sizes of genotype on AD, MD, and RD where an increase in purple denotes an increase in each dimension and pink denotes a decrease. **b** Boxplots of the mean intensity of AD, MD, and RD being significantly decreased in the inferior colliculus, rest of midbrain, and substantia nigra in *Fmr1-*^*Δ*^*exon 8*^−/−^ rats. Significance bars represent pair-wise comparisons from either a Tukey HSD or Dunn’s test, (*N* = 15/group), **p < 0.01 and *p < 0.05.

Contrary to the males, when we assessed these parameters in female WT, *Fmr1-*^*Δ*^*exon 8*^−/−^, and *Fmr1-*^*Δ*^*exon 8*^*−*^^*/*^^*+*^ littermates, we found that the brainstem was the most affected region. Genotype had a significant effect on AD, MD, and RD in the inferior colliculus (Fig. 4; ANOVA, *p* = 0.0022, *p* = 0.0061, and *p* = 0.021), substantia nigra (Fig. 4; ANOVA, *p* = 0.0048, *p* = 0.0057, and *p* = 0.0095), and the rest of the midbrain (Fig. 4; ANOVA, *p* = 0.29, *p* = 0.0059, and *p* = 0.01). Interestingly, there was a dose-dependent effect of Fmrp-^Δ^KH1 expression where AD, MD, and RD were highest in WT rats, lower in *Fmr1-*^*Δ*^*exon 8*^*−*^^*/*^^*+*^ rats, and lowest in *Fmr1-*^*Δ*^*exon 8*^−/−^ rats. AD, MD, and RD were all decreased in *Fmr1-*^*Δ*^*exon 8*^−/−^ compared to WT rats in the inferior colliculus (Fig. 4; Tukey HSD, *p adj*. = 0.0017, *p adj*. = 0.0043, and *p adj*. = 0.015). All three indices were similarly decreased in the substantia nigra in *Fmr1-*^*Δ*^*exon 8*^−/−^ compared to WT rats (Fig. 4; Tukey HSD, *p adj*. = 0.0051, *p adj*. = 0.005, and *p adj*. = 0.0082), and AD and MD were decreased in *Fmr1-*^*Δ*^*exon 8*^−/−^ compared to *Fmr1-*^*Δ*^*exon 8*^*−*^^*/*^^*+*^ rats (Fig. 4; Tukey HSD, *p adj*. = 0.03 and *p adj*. = 0.049). In the rest of the midbrain, which constituted everything in the midbrain that was not otherwise covered by the other specific regions in our analysis, all three indices were also lower in *Fmr1-*^*Δ*^*exon 8*^−/−^ compared to WT rats (Fig. 4; Tukey HSD, *p adj*. = 0.025, *p adj*. = 0.006, and *p adj*. = 0.012). MD and RD were decreased in *Fmr1-*^*Δ*^*exon 8*^−/−^ compared to *Fmr1-*^*Δ*^*exon 8*^*−*^^*/*^^*+*^ rats (Fig. 4; Tukey HSD, *p adj*. = 0.033 and *p adj*. = 0.039). Finally, a significant effect was observed in the amygdala, with increased FA in *Fmr1-*^*Δ*^*exon 8*^−/−^ compared to *Fmr1-*^*Δ*^*exon 8*^*−*^^*/*^^*+*^ rats (Fig. 4; Tukey HSD, *p adj*. = 0.01). This change in FA was not dose-dependent and the difference was largely due to the high variability in the *Fmr1-*^*Δ*^*exon 8*^−/−^ rats. This difference in FA could be driven by impairments to the myelin within the amygdala or neighboring voxels of white matter. Moreover, all of these effects in these gray matter regions could be driven by deficits in the minimal amount of axonal projections that exists within them or near them, especially in the afferent and efferent projections to these regions. Alternatively, they could be due to gray matter-specific deficits, such as increased spine head density. Notably, while AD, MD, and RD decreased in female *Fmr1-*^*Δ*^*exon 8*^−/−^ rats compared to WT controls, they increased in the male *Fmr1-*^*Δ*^*exon 8*^*−*^^*/*^^*y*^ rats. This raises the question of whether there would be an interaction between sex and genotype, which can only be addressed by comparing both in a single analysis.

### Combining sexes shows effect of genotype and uncovers major sex differences

We again validated our findings with a fully automated analysis that included both sexes (*Fmr1-*^*Δ*^*exon 8*^*−*^^*/*^^*+*^ rats were excluded as before) and found multiple regions to have altered microstructure due to genotype (Supplementary Tables 3 and 4). We discovered altered diffusion in the neocortex and inferior colliculus of *Fmr1-*^*Δ*^*exon 8* rats; both structures were impaired in male and female rats when they were individually compared to their sex-matched controls. FA was reduced in the anterior commissure, neocortex, and the rest of the forebrain in the *Fmr1-*^*Δ*^*exon 8* rats (Supplementary Fig. 4A; LM, *p* = 0.00039, *p* = 0.00086, and *p* = 0.00087, respectively). The finding that diffusivity in the anterior commissure is impaired further suggests that a decrease in *Fmr1* expression can lead to white matter impairments. In addition, we observed a substantial decrease in MD in the inferior colliculus in both sexes (Supplementary Fig. 4B; Tukey HSD, *p adj*. = 0.0013). Furthermore, RD was reduced in the amygdala (Supplementary Fig. 4C; LM, *p* = 0.00095) and hippocampus (Supplementary Fig. 4C; Tukey HSD, *p adj*. = 0.0015), again suggesting that there are deficits in the myelin within these gray matter regions or neighboring voxels of white matter, or changes to gray matter-specific cellular structures like dendritic spines.

**Fig. 5:**
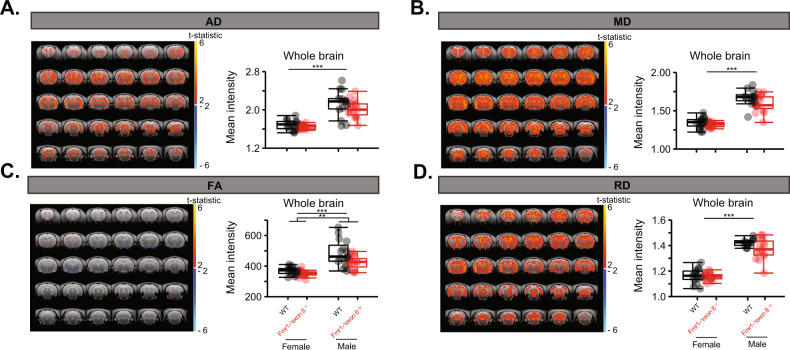
Increases in diffusion indices across the whole brain in male compared to female *Fmr1-*^*Δ*^*exon 8* and WT littermates. Maps of the t-statistic across the brain, showing the voxels that are increased or decreased in mean intensity in the male rats compared to the female rats and boxplots of group means across the whole brain for (**a**) AD, (**b**) MD, (**c**) FA, and (**d**) RD where the significance of the pair-wise comparisons from the Tukey HSD or LM is reported, (*N* = 15/group), ***p < 0.001 and **p < 0.01.

Unexpectedly, while there were no significant interactions between genotype and sex after correcting for multiple comparisons, there were highly significant effects of sex on these DTI metrics in each region where FA (Supplementary Fig. 4A; LM, *p* = 0.0029, *p* = 5.25 × 10^−5^, *p* = 7.71 × 10^−4^), MD (Supplementary Fig. 4B; Tukey HSD, *p adj*. 2.83 × 10^−10^), and RD parameters (Supplementary Fig. 4C; LM, *p* = 4.04 × 10^−9^ and Tukey HSD, *p adj*. < 1.0 × 10^−20^) were all higher in the males. All nominal *p* values survived a correction for multiple comparisons, except the *p* value for the effect of sex on FA in the anterior commissure and genotype on RD in the hippocampus. In fact, across the whole brain, males had much higher AD (Fig. 5a; LM, *p* = 7.45 × 10^−12^), MD (Fig. 5b; Tukey HSD, *p adj*. < 1.0 × 10^−20^), FA (Fig. 5c; LM, *p* = 5.07 × 10^−9^), and RD (Fig. 5d; Tukey HSD, *p adj*. < 1.0 × 10^−20^) than females, in which all nominal *p* values survived a correction for multiple comparisons. This could potentially explain the observed increase in diffusion in the male *Fmr1-*^*Δ*^*exon 8* rats and decrease in the female *Fmr1-*^*Δ*^*exon 8* rats compared to WT controls.

### Axon integrity is disrupted in the splenium of *Fmr1-*^*Δ*^*exon 8*^*−*^^*/*^^*y*^ rats

**Fig. 6:**
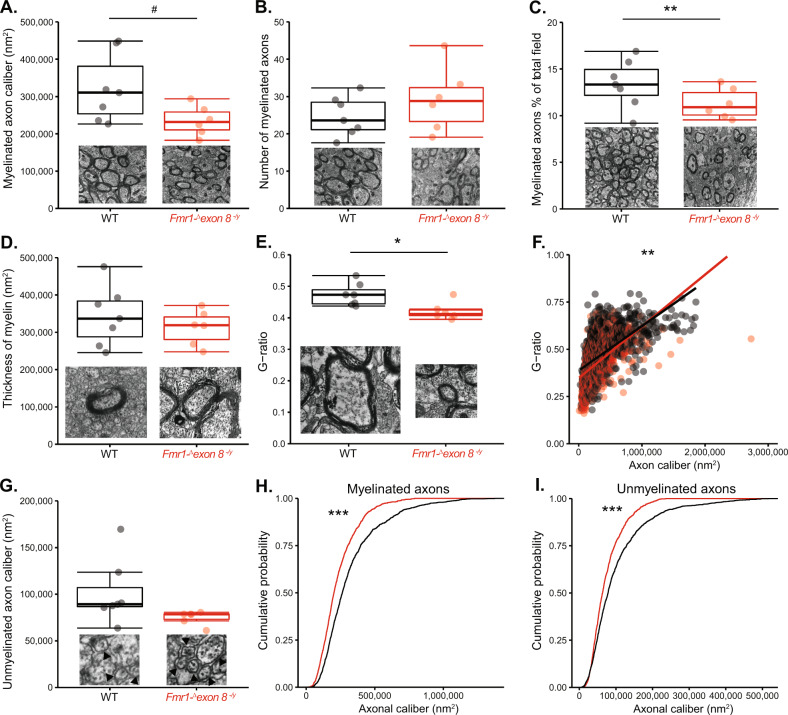
Ultrastructure of myelinated and unmyelinated axons in the splenium of the corpus callosum of male *Fmr1-*^*Δ*^*exon 8*^*−*^^*/*^^*y*^ and WT littermates. Boxplots of **a** the caliber and **b** number of myelinated axons, **c** the percentage of the total image that contains myelinated axons, **d** the thickness of the myelin, and **e** the g-ratio of myelinated axons. **f** The g-ratios compared to the caliber of the axons with their linear models. **g** Boxplot of axon caliber of unmyelinated axons, with arrows pointing to representative unmyelinated axons. Empirical distribution functions of **h** myelinated and **i** unmyelinated axons (WT: *N* = 7; *Fmr1-*^*Δ*^*exon 8*^*−*^^*/*^^*y*^: *N* = 6), ***p < 0.001, **p < 0.01, *p < 0.05, and ^#^*p* < 0.1. The insets are examples of the image fields or axons.

The brain-wide white matter deficits that followed a loss of *Fmr1-exon 8* expression were specific to male rats. The deficits to the splenium in the DTI analysis were particularly striking and may offer a potential locus for the impairments we previously observed in attention^11^ because it was increased in AD, MD, and RD in *Fmr1-*^*Δ*^*exon 8*^*−*^^*/*^^*y*^ rats. We examined it further at the level of its ultrastructure using electron microscopy, as the observed differences in diffusion metrics could be explained by changes at the ultrastructural level (Supplementary Table 3). We measured the caliber of the myelinated and unmyelinated axons, estimated the number of myelinated axons in our sample, and determined the thickness of the myelin in *Fmr1-*^*Δ*^*exon 8*^*−*^^*/*^^*y*^ rats and their sex-matched WT littermates. We found that the caliber of myelinated axons was trending to be significantly reduced in *Fmr1-*^*Δ*^*exon 8*^*−*^^*/*^^*y*^ rats (Fig. 6a; LM, *p* = 0.056). The reduced caliber of these myelinated axons could restrict diffusion along the axis of the axon and underlie the increase in AD seen with DTI in the *Fmr1-*^*Δ*^*exon 8*^*−*^^*/*^^*y*^ rats. Furthermore, while there was no difference in the number of myelinated axons per field (Fig. 6b; LM, *p* = 0.14), when we multiplied the mean caliber of the myelinated axons by the number represented in a given field, we found that the myelinated axons occupied significantly less of the total field area in *Fmr1-*^*Δ*^*exon 8*^*−*^^*/*^^*y*^ rats (Fig. 6c; LM, *p* = 0.0012). This suggests that there is more room for diffusion outside of the myelinated axons, which could lead to an increase in MD and RD. However, it is unclear whether MRI is sensitive enough to capture these types of changes. Notably, we did not see deficits in myelin integrity. The thickness of the myelin was roughly equal in male WT rats and *Fmr1-*^*Δ*^*exon 8*^*−*^^*/*^^*y*^ rats (Fig. 6d; LM, *p* = 0.28). Interestingly, however, the mean g-ratio was reduced in the *Fmr1-*^*Δ*^*exon 8*^*−*^^*/*^^*y*^ rats (Fig. 6e; LM, *p* = 0.02). To assess whether reduced axonal caliber could be driving this decrease, we examined the relationship between g-ratio and axon caliber. We found a significant interaction between genotype and axon caliber where smaller axons had decreased g-ratios in *Fmr1-*^*Δ*^*exon 8*^*−*^^*/*^^*y*^ rats (Fig. 6f; LM, *p* = 0.0012). These lower g-ratios in the smaller axons could be due to thicker myelin or reduced internode length. In addition, thicker myelin surrounding the smaller axons could further restrict diffusion along these axons.

Unlike myelinated axons, the caliber of unmyelinated axons was unaffected in the *Fmr1-*^*Δ*^*exon 8*^*−*^^*/*^^*y*^ rats (Fig. 6g; LM, *p* = 0.1). However, interestingly, when we evaluated the proportion of axons at each caliber, we found a significant difference comparing the cumulative frequencies of axonal calibers in the *Fmr1-*^*Δ*^*exon 8*^*−*^^*/*^^*y*^ rats compared to their WT littermates of both myelinated and unmyelinated axons (Fig. 6h, i; Kolmogorov–Smirnov test, *p* < 2.2 × 10^−16^ and *p* < 9.78 × 10^−11^, *D* = 0.19 and *D* = 0.14). These frequency plots show that the fields from the *Fmr1-*^*Δ*^*exon 8*^*−*^^*/*^^*y*^ rats contained smaller myelinated (< 750 000 nm^2^) and unmyelinated (~50,000 to ~250,000 nm^2^) axons and did not contain larger (> 750 000 nm^2^) myelinated and unmyelinated (>250,000 nm^2^) axons that were present in the WT images. Overall, the impairments in the splenium in *Fmr1* KO mice that were discovered using DTI appear to be at least partially explained by a general reduction in axonal caliber.

## Discussion

In this study, we discovered structural perturbations in several brain regions of *Fmr1-*^*Δ*^*exon 8* rats, including in specific domains of white matter, and decreased caliber of axons in the splenium of the corpus callosum. Therefore, a loss of function of the KH1 domain of Fmrp and/or low expression of Fmrp is able to cause large-scale anatomical dysmorphology that might follow from dysregulated expression of mRNA targets. In white matter, this may result from reductions in axonal integrity. Importantly, we discovered volumetric changes and alterations in diffusion in both gray and white matter regions that are also impaired in *Fmr1* KO mice and individuals with FXS, as detailed below. Furthermore, all of these anatomical perturbations could be related to the deficits we see in behavior and transcription of *Fmr1-*^*Δ*^*exon 8* rats^11^. This relationship is worth further exploration.

Given that *FMR1* is X-linked, FXS affects male individuals more often than female individuals, the latter being predominantly heterozygous in expression of the *Fmr1* mutation^2^. Uniquely, with a rodent model, we were able to generate females that express the mutation homozygously. This allowed us to discover that, just as both male and female *Fmr1-*^*Δ*^*exon 8* rats exhibit severe sustained attention deficits^11^, the loss of *Fmr1-exon 8* expression leads to structural impairments in both sexes without an interaction with sex. Furthermore, because only females can be heterozygous in the expression of the mutation, including female rats in our analysis allowed us to probe for dose-dependent effects of Fmrp-^Δ^KH1 expression. We found a dose-dependent effect of Fmrp-^Δ^KH1 on AD, MD, and RD in the substantia nigra and in the rest of the midbrain. Our findings suggest that future preclinical FXS studies should consider the inclusion of females. Female individuals with FXS, although rarer in the population, are also in need of treatment and should not be overlooked. Including females in our study design also led to the discovery of a general increase in diffusion across the brain in males compared to females, regardless of genotype. A similar trend was also identified for FA and AD in humans^24^. These authors attributed this sex difference to hormonal exposure, but it could also be due to sex differences in the trajectories of neurological development and microstructural maturation. It has also been shown that males have greater within-hemisphere connectivity and females have greater between-hemisphere connectivity, which could potentially affect these measures^25^. The sex difference in these rats should be replicated in another strain.

Many of our current findings replicate those from full *Fmr1* KO mice and individuals with FXS, thus building upon our previous conclusion that the *Fmr1-*^*Δ*^*exon 8* rat is a valid model of FXS^11^. We determined that the volumes of the superior colliculus, genu of the corpus callosum, hypothalamus, and PAG were impaired following a loss of expression of the KH1 domain. The superior colliculus, hypothalamus, and PAG are all also impaired in *Fmr1* KO mice^5,6^ and the genu of the corpus callosum is increased in overall diffusion in girls with FXS^4^. Notably, the superior colliculus and PAG have not garnered much attention in studies of individuals with FXS and their volume in this population is under-reported. Considering that they are affected in both a mouse and a rat model of FXS, crucially in both sexes of a rat model, their structure should be assessed in individuals with FXS.

We further found alterations in diffusion in both gray and white matter regions that are also impaired in *Fmr1* KO mice and individuals with FXS. In the forebrain, for example, the caudate/putamen and internal capsule are similarly smaller and the fimbria and inferior colliculus are similarly enlarged in the *Fmr1* KO mouse on the FVB background strain compared to WT controls^5^. This reduction in size of the caudate nucleus is opposite of what is often documented in individuals with FXS, but the caudate and putamen are separate nuclei in humans, making them difficult to compare to their rodent homolog. Unlike the caudate nucleus, the fimbria and inferior colliculus have not been explored as regions that could underlie FXS pathology. The anterior limbs of the internal capsule are increased in MD and RD in adolescents with FXS^4^, which complements what we found in our *Fmr1-*^*Δ*^*exon 8*^*−*^^*/*^^*y*^ rats. Further complementing our results, the splenium and thalamus show increased AD, MD, and RD in girls with FXS^4^, the thalamus has increased gray matter volume^26^, and FA is decreased in the splenium of *Fmr1* KO mice^7^. Although FA is often disrupted in humans with FXS and *Fmr1* KO mouse models^4,7^, it was the parameter with the least degree of variation in our rats. Interestingly, no prior studies have examined the anatomy of the substantia nigra in FXS even though dopaminergic tone is dampened^27^, likely because this dysfunction is most often attributed to deficits in the frontostriatal pathway that are often observed in FXS. An increase in MD in the substantia nigra typically indicates a loss of dopaminergic neurons in Parkinson’s disease^28^. *Fmr1* KO mice have fewer tyrosine hydroxylase-expressing neurons in the substantia nigra^29^. The decrease in diffusion that we observed could potentially be related to the integrity of the dopaminergic neurons in *Fmr1-*^*Δ*^*exon 8*^−/−^ rats.

Many brain regions we found to be altered in structure are also impaired in FXS, are involved in attention networks, and are implicated in ADHD. These structures are worth exploring further as targets for treatment of attention deficits in FXS. For example, the superior colliculus is implicated in visuospatial attention across species^30^, most specifically in spatial orienting, and the caudate and putamen are known to be implicated in impulsivity^31^. In ADHD, MD in the caudate, putamen, and thalamus is correlated with increased reaction time on a flanker test^32^. Furthermore, the internal capsule has impaired white matter integrity^33^. Lastly, there is higher MD and RD, and lower FA^34^ in the splenium. The lower FA phenotype is especially prominent in patients with the inattention phenotype^33^, the specific deficit we discovered in the *Fmr1-*^*Δ*^*exon 8* rats^11^. Therefore, the relationship between the altered structure of these regions and attentional functioning in FXS models should be studied more directly in the same animals.

Sensory deficits have also been replicated in FXS models^35^ and many of the brain regions that were perturbed in our study are implicated in sensory perception. To begin with, three regions that are involved in olfaction, the piriform cortex, olfactory bulb, and anterior commissure were altered in *Fmr1-*^*Δ*^*exon 8* rats. Olfaction has not been well characterized in FXS, but it is impaired in rodent models of FXS^36^. Individuals with FXS can also be hypersensitive to auditory and visual stimuli. We found that the inferior and superior colliculus, which form part of the auditory and visual pathways respectively, are altered in *Fmr1-*^*Δ*^*exon 8* rats. In addition, we identified alterations in the PAG, which, among other functions, is involved in somatosensory perception and the thalamus, which relays sensory information to the cortex, to have increased diffusion. C-fos expression in the PAG and thalamus is excessive after an auditory stimulus that causes audiogenic-induced seizures in *Fmr1* KO mice^37,38^. The role that these regions play in hypersensitivity in FXS has not garnered much attention. The connection between the structural alterations we identified and the sensory deficits that are often observed in FXS models should be further explored.

The splenium of the corpus callosum showed increases in all three measures of diffusion. Because the splenium is related to attention and this finding recapitulates what is seen in girls with FXS, we decided to explore its integrity further at the level of histology. Using electron microscopy, we found that axons were decreased in caliber, myelinated axons occupied less of the image, and smaller axons had thicker myelin in *Fmr1-*^*Δ*^*exon 8*^*−/y*^ rats. It is possible that the reduced caliber of these axons and the increased thickness of myelin surrounding smaller axons could restrict diffusion along the axis of the axon, contributing to the increase in AD we identified with DTI. It is further possible that because the myelinated axons inhabited less of the image, there could be more room for MD and RD. This association warrants further study. Regardless of the relationship, this is the first time that axon caliber was assessed in the corpus callosum in a rat model of FXS. This again replicates findings from a full *Fmr1* KO mouse, which showed smaller axon diameters in the corpus callosum^39^. This raises the question of whether the reduction that was discovered in the corpus callosum was due to reductions in the splenium. Axonal caliber can depend upon the amount of energy available, which is mostly regulated by mitochondrial function^40^. In the immature neurons of the dentate gyrus of adult *Fmr1* KO mice, mitochondria are shorter and mitochondria-related genes show decreased expression^41^. Furthermore, mitochondrial membrane potential in cultured neurons is reduced and oxidative stress is increased, indicating mitochondrial dysfunction. It should be assessed whether the same phenomena could be occurring in the axons of the splenium of *Fmr1-*^*Δ*^*exon 8*^*−/y*^ rats, which could offer a potential mechanism for the reduction in axonal caliber we identified. Interestingly, we previously found that PGC-1α (also known as PPARGC1A), a master regulator of mitochondria biogenesis that is also an Fmrp target, is decreased in expression in *Fmr1-*^*Δ*^*exon 8*^−^^*/y*^ rats^11^. It would be worthwhile to determine whether PGC-1α plays a role in this deficit in axonal caliber. Weakening of the cytoskeleton could also cause a reduction in caliber. Fmrp regulates the translation of mRNAs that encode for proteins that regulate cytoskeleton remodeling. Fmrp localizes to axons and associates with translational machinery in rats^42^. It is, therefore, possible that the expression of proteins that regulate the integrity of the cytoskeleton is dysregulated in *Fmr1-*^*Δ*^*exon 8*^−*/y*^ rats, leading to malformed axonal cytoskeleton.

In summary, we have shown here that a specific deletion of exon 8 of the *Fmr1* gene is sufficient to cause FXS-like changes in neuroanatomy in rat, specifically in the axons of a major pathways. Therefore, these affected regions and the white matter within them deserve further examination and could serve as potential cross-species non-invasive biomarkers for FXS.

## Supplementary information

Supplementary Figure 1

Supplementary Figure 2

Supplementary Figure 3

Supplementary Figure 4

Supplementary Figures

Supplementary Table 1

Supplementary Table 2

Supplementary Table 3

Supplementary Table 4
