# Supplementary material for: Reduced axonal caliber and structural changes in a rat model of Fragile X syndrome with a deletion of a K-Homology domain of Fmr1
Source: Transl Psychiatry. 2020 Aug 12;10:280. doi: 10.1038/s41398-020-00943-x (PMC7423986; doi:10.1038/s41398-020-00943-x)
Supplement: Supplementary file 5 — Supplementary Figures [file 41398_2020_943_MOESM5_ESM.pdf]

Supplementary Figure 1. Increased absolute volume of the superior colliculus in male *Fmr1-Δexon 8*$^{-/y}$ rats compared to WT littermates. (A) Heatmap of effect sizes of genotype on significant differences in absolute volume in T2 images where an increase in purple denotes an increase in *Fmr1-Δexon 8*$^{-/y}$ compared to WT rats and pink denotes a decrease. **(B)** Boxplot of the significantly increased volume of the superior colliculus, (WT: N = 15; *Fmr1-Δexon 8*$^{-/y}$: N = 14).

Supplementary Figure 2. Volumes of brain regions of male and female *Fmr1-Δexon 8* and WT littermates. (A) Heatmap of *p*-values from ANOVA and LM tests for the effect of sex on volume in T2 images where an increase in red denotes increased Bonferroni-corrected significance. **(B)** Boxplot of the volume of the whole brain, (N = 15/group), ***$p < 0.001$.

Supplementary Figure 3. Example coronal slices of the regions that were altered in *Fmr1-Δexon 8* rats, using the semi-manual segmentation and Waxholm Space Atlas.

Supplementary Figure 4. Voxel-wise analysis of diffusion indices in male and female *Fmr1-Δexon 8* and WT littermates. Maps of the t-statistic across the brain, showing the voxels that are increased or decreased in mean intensity in the *Fmr1-Δexon* rats compared to WT and boxplots of group means for **(A)** FA, **(B)** MD, and **(C)** RD where the significance of the pair-wise comparisons from the Tukey HSD or LM is reported, (N = 15/group), ***$p < 0.001$.
